# Assessing heterogeneity of patient and health system delay among TB in a population with internal migrants in China

**DOI:** 10.3389/fpubh.2024.1354515

**Published:** 2024-02-02

**Authors:** Ruoyao Sun, Zheyuan Wu, Hongyin Zhang, Jinrong Huang, Yueting Liu, Meiru Chen, Yixiao Lv, Fei Zhao, Yangyi Zhang, Minjuan Li, Jiaqi Yan, Hongbing Jiang, Yiqiang Zhan, Jimin Xu, Yanzi Xu, Jianhui Yuan, Yang Zhao, Xin Shen, Chongguang Yang

**Affiliations:** ^1^School of Public Health (Shenzhen), Shenzhen Campus, Sun Yat-sen University, Shenzhen, Guangdong Province, China; ^2^Division of TB and HIV/AIDS Prevention, Shanghai Municipal Center for Disease Control and Prevention, Shanghai, China; ^3^Shanghai Institutes of Preventive Medicine, Shanghai, China; ^4^Department of Pharmacy, Beijing Hospital, National Center of Gerontology; Institute of Geriatric Medicine, Chinese Academy of Medical Sciences; Beijing Key Laboratory of Assessment of Clinical Drugs Risk and Individual Application (Beijing Hospital), Beijing, China; ^5^Department of Epidemiology, School of Public Health and Key Laboratory of Public Health Safety, Fudan University, Shanghai, China; ^6^Nanshan District Center for Disease Control and Prevention, Shenzhen, Guangdong Province, China; ^7^Department of Epidemiology of Microbial Diseases, Yale School of Public Health, New Haven, CT, United States

**Keywords:** tuberculosis, TB diagnosis delay, patient delay, health system delay, Bayesian spatial analysis

## Abstract

**Backgrounds:**

The diagnostic delay of tuberculosis (TB) contributes to further transmission and impedes the implementation of the End TB Strategy. Therefore, we aimed to describe the characteristics of patient delay, health system delay, and total delay among TB patients in Shanghai, identify areas at high risk for delay, and explore the potential factors of long delay at individual and spatial levels.

**Method:**

The study included TB patients among migrants and residents in Shanghai between January 2010 and December 2018. Patient and health system delays exceeding 14 days and total delays exceeding 28 days were defined as long delays. Time trends of long delays were evaluated by Joinpoint regression. Multivariable logistic regression analysis was employed to analyze influencing factors of long delays. Spatial analysis of delays was conducted using ArcGIS, and the hierarchical Bayesian spatial model was utilized to explore associated spatial factors.

**Results:**

Overall, 61,050 TB patients were notified during the study period. Median patient, health system, and total delays were 12 days (IQR: 3–26), 9 days (IQR: 4–18), and 27 days (IQR: 15–43), respectively. Migrants, females, older adults, symptomatic visits to TB-designated facilities, and pathogen-positive were associated with longer patient delays, while pathogen-negative, active case findings and symptomatic visits to non-TB-designated facilities were associated with long health system delays (LHD). Spatial analysis revealed Chongming Island was a hotspot for patient delay, while western areas of Shanghai, with a high proportion of internal migrants and industrial parks, were at high risk for LHD. The application of rapid molecular diagnostic methods was associated with reduced health system delays.

**Conclusion:**

Despite a relatively shorter diagnostic delay of TB than in the other regions in China, there was vital social-demographic and spatial heterogeneity in the occurrence of long delays in Shanghai. While the active case finding and rapid molecular diagnosis reduced the delay, novel targeted interventions are still required to address the challenges of TB diagnosis among both migrants and residents in this urban setting.

## 1 Introduction

Tuberculosis (TB) is one of the major infectious diseases threatening global public health and the leading cause of morbidity from a single infectious agent worldwide (1). China has made significant progress in TB control over the past decade (2). However, according to the WHO Global Tuberculosis Report 2022, there were approximately 780,000 new TB cases in China (1), indicating it still faces an enormous challenge in achieving the key goals of the End TB Strategy in 2035.

To reduce the global burden of TB, the WHO recommends a key next step of prioritizing interventions for early detection and diagnosis (3). However, TB is usually asymptomatic or presents with non-specific symptoms in the early stages (4). Most countries adopt passive case finding as the predominant pathway to TB case detection (3), meaning that the disease may have progressed for weeks when patients first visit a health facility. Besides, some health systems cannot make rapid diagnoses due to their facilities or lack of capacity to diagnose the disease (5). Therefore, many countries have failed to detect TB in time, resulting in long delays, which can lead to further disease progression and unfavorable outcomes. Furthermore, delayed diagnosis allows TB to continue to spread within the community (6). Thus, diagnostic delay is a major problem for TB control programs, especially in high-burden countries.

Several studies have focused on TB diagnostic delay, exploring the potential risk factors. Age, gender, education, occupation, low income, and HIV co-infection were reported to affect delays (7–10). Besides, seeking care in low-level facilities and smear-negative can cause longer diagnostic delays (11). Moreover, a study in Portugal identified high delay areas and associated ecological factors (12). Although there have been some studies on TB diagnostic delays (13–15), the risk factors of long delays can differ based on the TB epidemiological characteristics of the study region, and the spatial heterogeneity of long diagnostic delay was unclear. In China, a national study showed that more than 50 percent of TB patients were not diagnosed in time (16), suggesting that TB delay was still common in China. This issue necessitates the implementation of targeted control measures.

With the accelerated urbanization in the past decades of China, numerous migrants from other provinces have moved to major cities like Shanghai to seek better job opportunities (17). The large population of migrants has brought a considerable challenge in the timely diagnosis of TB. However, the delays and associated factors among TB patients have not been adequately investigated. Therefore, we aimed to characterize the patient, health system, and total delay of TB patients in Shanghai, describe the time trends of the delays, identify high delay areas, and analyze the factors associated with long delays at both individual and regional levels.

## 2 Materials and methods

### 2.1 Study setting and data collection

Shanghai is one of China’s megacities, with an estimated population of more than 24 million, of which over 40% are migrants (17). It comprises 16 districts and 214 counties. Shanghai has a well-established TB service management system. All patients suspected of having TB are referred to TB-designated hospitals, where the diagnosis is confirmed. Healthcare staff or nurses at TB-designated hospitals use a series of standard questions and forms to collect information on the diagnosis, treatment, and management of confirmed patients, which is then recorded in the TB surveillance system and reported to the Shanghai Municipal Center for Disease Control and Prevention (Shanghai CDC) (18).

We included TB patients diagnosed between January 1st, 2010, and December 31st, 2018, in Shanghai. We included cases up to 2018 because of the potential impact of COVID-19, which began in late 2019, on tuberculosis-related data. Patients with missing symptom onset dates, first medical visit dates, or diagnosis dates, and those diagnosed with extrapulmonary tuberculosis, tuberculous pleurisy, or non-tuberculous mycobacteria, and patients with unknown address information were excluded from the analysis. We collected the following information through a standard procedure, including the gender, age, demographic attributes, ethnicity, occupation, current address, patient source, severe case, TB history, and pathogen testing results of all confirmed TB patients.

### 2.2 Definitions

According to the WHO definition, patient delay is defined as the time interval between the onset of symptoms and the first visit to a health facility. Health system delay is the interval between the first visit to a health facility and the diagnosis of TB (19). The total delay is the sum of patient and health system delays. Based on previous studies and the national TB control program in China (11, 14, 20, 21), long patient delay (LPD) and long health system delay (LHD) were defined as delay days exceeding 14 days. Long diagnostic delay (LDD) was defined as a total delay longer than 28 days.

According to the household registration system in China, people with Shanghai household registration were identified as residents, whereas those without Shanghai household registration were classified as migrants. Severe cases can be defined as active tuberculous lesions in the lung tissue, trachea, bronchi, and pleura, with severe hypoxemia or acute respiratory failure requiring mechanical ventilation, or with hypotension, shock, and other signs of circulatory failure and other organ dysfunction.

The active pulmonary tuberculosis diagnosis was mainly based on the national diagnostic criteria for TB (22). Patients with any positive sputum smear, sputum culture, or molecular test were defined as pathogen-positive, and patients with negative sputum smear, sputum culture, and molecular test were defined as pathogen-negative TB patients.

The patient source was the way of discovering patients, and it was classified into the following categories (23): (1) Symptomatic visits to TB-designated facilities: Patients with suspected TB symptoms sought care at TB-designated facilities. (2) Symptomatic visits to non-TB-designated facilities: Patients with suspected TB symptoms sought care at non-TB-designated facilities and then were referred to TB-designated facilities. (3) Physical examination: Patients are identified and referred through health screening. (4) Close contact tracing: Patients identified through contact tracing for a known case. (5) Tracking after referrals: Tracking by the CDC of suspected TB patients who are not referred to designated facilities promptly. Besides, in the current study, ‘active case finding (ACF)’ included physical examination and close contact tracing.

### 2.3 Spatial analysis and Bayesian spatial modeling

We used the Google Maps tool to geocode the residential addresses and used Baidu Maps tool to verify or manually correct addresses that could not be recognized. We used ArcGIS 10.2 (ESRI Inc., Redlands, CA, United States) to describe the spatial distribution of diagnostic delay days (24).

The Getis-Ord Gi* method was used to identify the spatial clustering areas of long delay in Shanghai. Z-scores greater than or equal to 1.96 and *p*-values less than 0.05 indicate significant hotspot areas (25).

To further explore the long delay areas and influencing factors, we used the hierarchical Bayesian spatial model for the analysis. The model is as follows:


$$Y_{k}|\mu_{k}\sim f\left(y_{k}|\mu_{k},\nu^{2}\right);k=1,\ldots ,n\text{,}$$

$$g\left(\mu_{k}\right)=x_{k}^{T}\beta +\phi_{k}+O_{k}$$

Y_k_ is the actual number of long delay cases in region k. μ_k_ is the expected number of long delay cases. O_k_ is a vector of known offsets. x_k_ is the vector of county-level predictors of long-delay risk. ϕ_k_ is the random effect, including spatial effects. β is the coefficient of covariate. ν^2^ is an additional scale parameter required if the Gaussian family is used (26).

We used the R package “CARBayes” to implement the model and selected the Leroux model to reveal the overall spatial random effect. We adopted the conditional autoregressive (CAR) prior for the spatial structure, with a binary spatial weight matrix W. Based on previous research and data availability (25, 27), we extracted county-level information from official databases, including the Shanghai Municipal Statistics Bureau and Shanghai Health Commission. The variables in the model were comprised of population density, *per capita* GDP, the proportion of migrants, household size, total sex ratio (the number of males compared to the number of females), proportion of older adult population, average annual TB notification rate, healthcare staff, and the presence of industrial areas. We used Markov chain Monte Carlo (MCMC) to simulate the model and calculate the relative risk and 95% credible interval (RR and 95%CI) of the posterior distribution. We also used the Bayesian spatial disease mapping method to analyze high-risk areas with long delays.

### 2.4 Statistical analysis

Patient characteristics were described using frequencies and proportions. Median and interquartile range (IQR) were used to describe the days of delay. We used the Join-point regression model to analyze temporal trends of delay. We used the survival analysis to estimate the probability of TB patients visiting a health facility or being diagnosed by delay status, and the Kaplan–Meier estimator to plot survival curves (28). We explored factors associated with LPD, LHD, and LDD by univariate and multivariable logistic regression analysis. The statistical analysis was performed using R (version 4.2.1), SPSS (version 22.0), and Join-point software (version 5.0.2). A value of *p* of <0.05 was considered statistically significant.

## 3 Results

### 3.1 Baseline characteristics of TB patients and the pattern of diagnosis delay

A total of 61,050 TB patients were included in the study (Supplementary Figure S1), and the sociodemographic and clinical characteristics are shown in Table 1. Migrants made up 44.2% of patients. More than two-thirds of patients (68.4%) were male. The main patient sources were symptomatic visits to TB-designated facilities (54.0%), followed by Symptomatic visits to non-TB-designated facilities (36.5%). In addition, 50.3% of patients were pathogen-positive.

**Table 1:** Characteristics of TB patients and median delay days stratified by different variables.

Characteristics	n (%)	Patient delay	Health system delay	Total delay
Demographic attributes				
Residents	34,081 (55.8)	13 (4–26)	9 (4–18)	27 (15–43)
Migrants	26,969 (44.2)	10 (2–26)	9 (4–19)	26 (14–43)
Gender				
Male	41,752 (68.4)	11 (3–25)	9 (4–18)	26 (14–42)
Female	19,298 (31.6)	13 (4–27)	9 (4–19)	28 (15–45)
Age				
<15	248 (0.5)	14 (2–27)	8.5 (4–15)	26 (14–45)
15–24	11,330 (18.6)	10 (2–24)	9 (5–18)	24 (14–40)
25–44	21,433 (35.1)	11 (3–26)	9 (4–19)	26 (14–42)
45–64	17,204 (28.2)	13 (4–27)	9 (4–18)	28 (15–45)
≥65	10,799 (17.7)	13 (5–27)	9 (5–19)	28 (15–46)
Ethnic group				
Han Chinese	60,543 (99.2)	12 (3–26)	9 (4–18)	27 (15–43)
Non-Han minority	5.7 (0.8)	10 (2–29)	9 (5–18)	26 (15–43)
Occupations				
Commercial service	3,533 (5.8)	9 (2–25)	10 (5–20)	27 (15–43)
Labor workers	10,660 (17.5)	8 (2–26)	10 (5–19)	25 (14–44)
Farmers	2,972 (4.9)	12 (3–30)	9 (4–18)	28 (15–50)
Students/Adolescents	2,893 (4.7)	10 (2–23)	9 (5–16)	23 (13–39)
Retirement	13,213 (21.6)	13 (5–26)	9 (3–19)	27 (17–44)
Household/Unemployed	8,459 (13.9)	10 (2–28)	9 (5–20)	27 (14–46)
Others	14,916 (24.4)	14 (5–25)	8 (2–17)	26 (15–40)
Unknown	4,404 (7.2)	15 (5–27)	9 (4–20)	30 (17–45)
Patient source				
Symptomatic visits to TB-designated facilities	32,988 (54.0)	15 (6–27)	8 (2–16)	28 (16–43)
Symptomatic visits to non-TB-designated facilities	22,223 (36.5)	8 (2–24)	10 (5–19)	24 (14–42)
Physical examination	1917 (3.1)	0 (0–1)	15 (8–27)	19 (10–34)
Close contact tracing	48 (0.1)	0 (0–4.5)	16 (6–20)	20 (8–33)
Tracking after referrals	3,806 (6.2)	10 (3–30)	13 (7–27)	31.5 (18–58)
Others	68 (0.1)	16 (0–42)	16.5 (5–54)	48.5 (21–90)
Severe cases				
No	45,716 (74.9)	12 (3–26)	10 (5–20)	28 (16–46)
Yes	15,334 (25.1)	13 (4–26)	10 (5–19)	28 (16–43)
Treatment category				
New	55,087 (90.2)	12 (3–26)	9 (4–18)	26 (14–43)
Retreated	5,963 (9.8)	12 (4–26)	10 (5–19)	28 (16–43)
Pathogen result				
Positive	30,704 (50.3)	12 (3–26)	8 (4–15)	26 (14–44)
Negative	28,871 (47.3)	11 (3–24)	11 (5–21)	27 (16–43)
Unknown	1,475 (2.4)	15 (6–26)	5 (0–13)	25 (14–36)

*delays are given as Median days (IQR).

**Figure 1:**
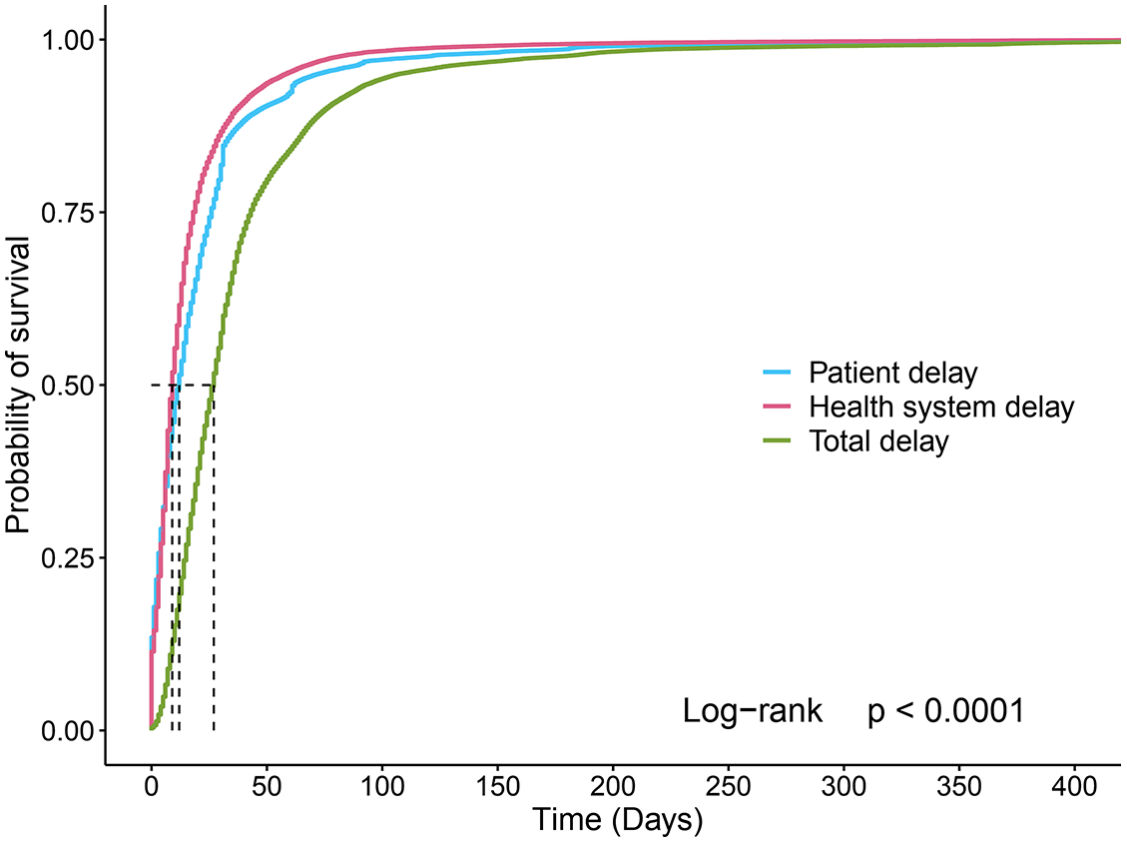
Survival curves for the patient, health system, and total delays. For patient delay curves, the graph shows the probability of visiting a health facility at different time points. The graph shows the probability of being diagnosed at different time points for health system delay and total delay curves.

Figure 1 shows the survival curves for patient, health system, and total delay. The delay curves were similar in pattern, with the initial curve growing rapidly and more patients arriving at the event quickly, and then the curve slowing over time. Overall, the median total delay day was 27 days (IQR: 15–43), the median patient delay was 12 days (IQR: 3–26), and the median health system delay was 9 days (IQR: 4–18). A total of 46.4% (28,319/61050) of the patients had LDD, 43.9% had LPD, and 32.3% had LHD.

### 3.2 Time trends of delay between 2010 and 2018

**Figure 2:**
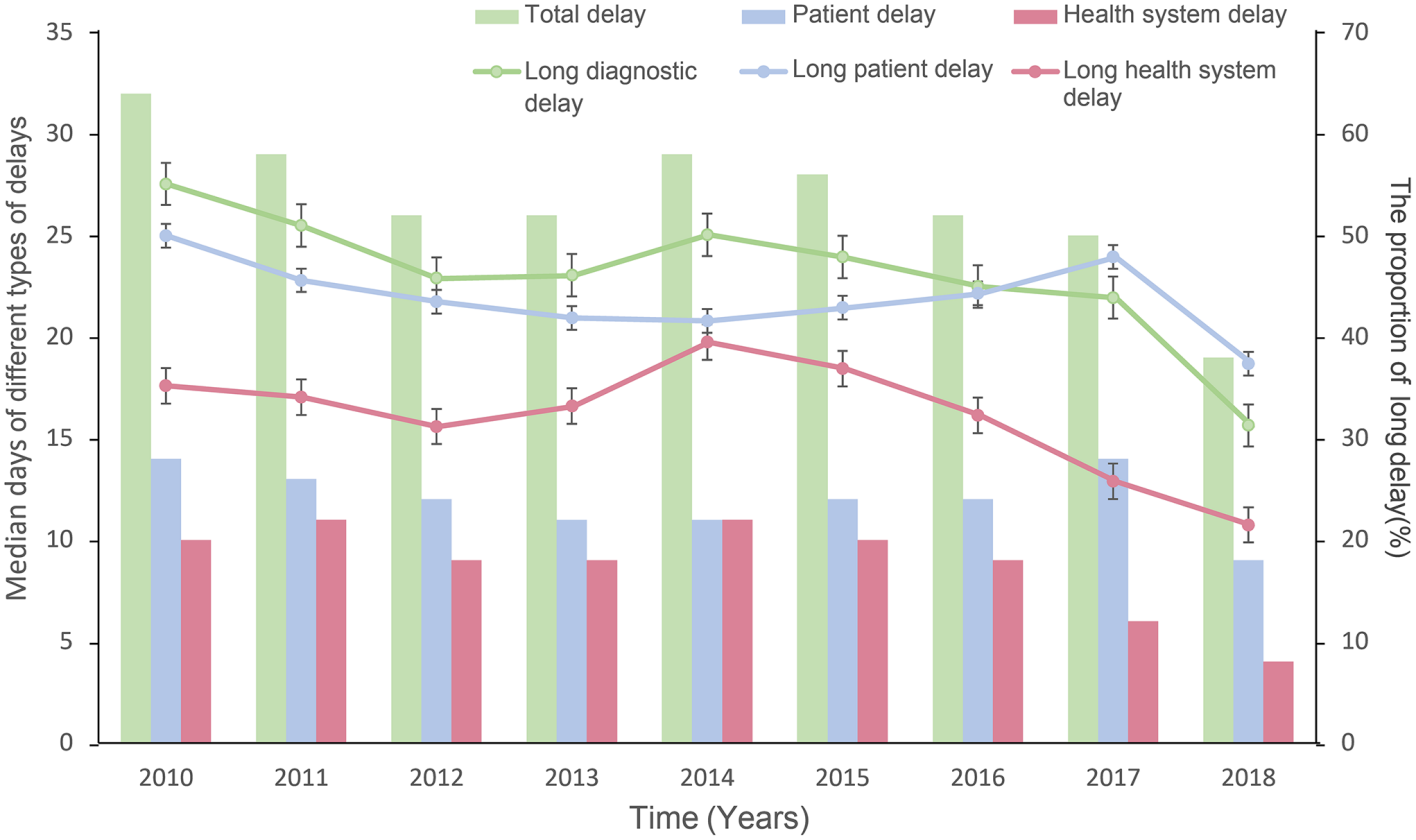
Median days of different types of delays and the proportion of long delays with confidence intervals (95%CI) by patient, health system, and total delay. The bar graph represents the median days of patient, health system, and total delay. The line graph represents the proportion of patients with long delays in the total number of TB patients.

Between 2010 and 2018, the total delay showed a decreasing trend of 13 days (32 to 19 days). However, the median time of patient delay did not change significantly (14 to 9 days). The median time of health system delay decreased significantly from 10 days in 2015 to 4 days in 2018. The trends for LDD, LPD, and LHD were similar to those for the median time of delay. The proportion of LDD decreased from 55.1% in 2010 to 31.3% in 2018, and LHD from 35.2% in 2010 to 21.5% in 2018 (Figure 2).

The joinpoint regression also showed the temporal trends of the proportion for long delays (Supplementary Figures S2A–C). We found a significant decrease in LDD between 2010 and 2018 (APC = −3.91, *p* = 0.017). There was no significant change in the trend of LPD (APC = −1.46, *p* = 0.184). For LHD, we found a turning point in 2015 (APC = 2.52, *p* = 0.380, 2010–2015; APC = -16.55, *p* = 0.057, 2015–2018), and the trend of LHD has decreased since 2015. The time trends for long delays in both migrants and residents were consistent with the total population (Supplementary Figures S2D–I). The proportion of LDD showed a decreasing trend (residents: APC = −1.74, *p* = 0.011; migrants: APC = −1.09, *p* = 0.049), with LHD having a turning point in 2015 (residents: APC = 0.25, *p* = 0.918, 2010–2015; APC = −18.20, *p* = 0.039, 2015–2018; migrants: APC = 5.55, *p* = 0.156, 2010–2015; APC = −14.98, *p* = 0.104, 2015–2018).

### 3.3 Individual factors associated with long delay of TB

**Figure 3:**
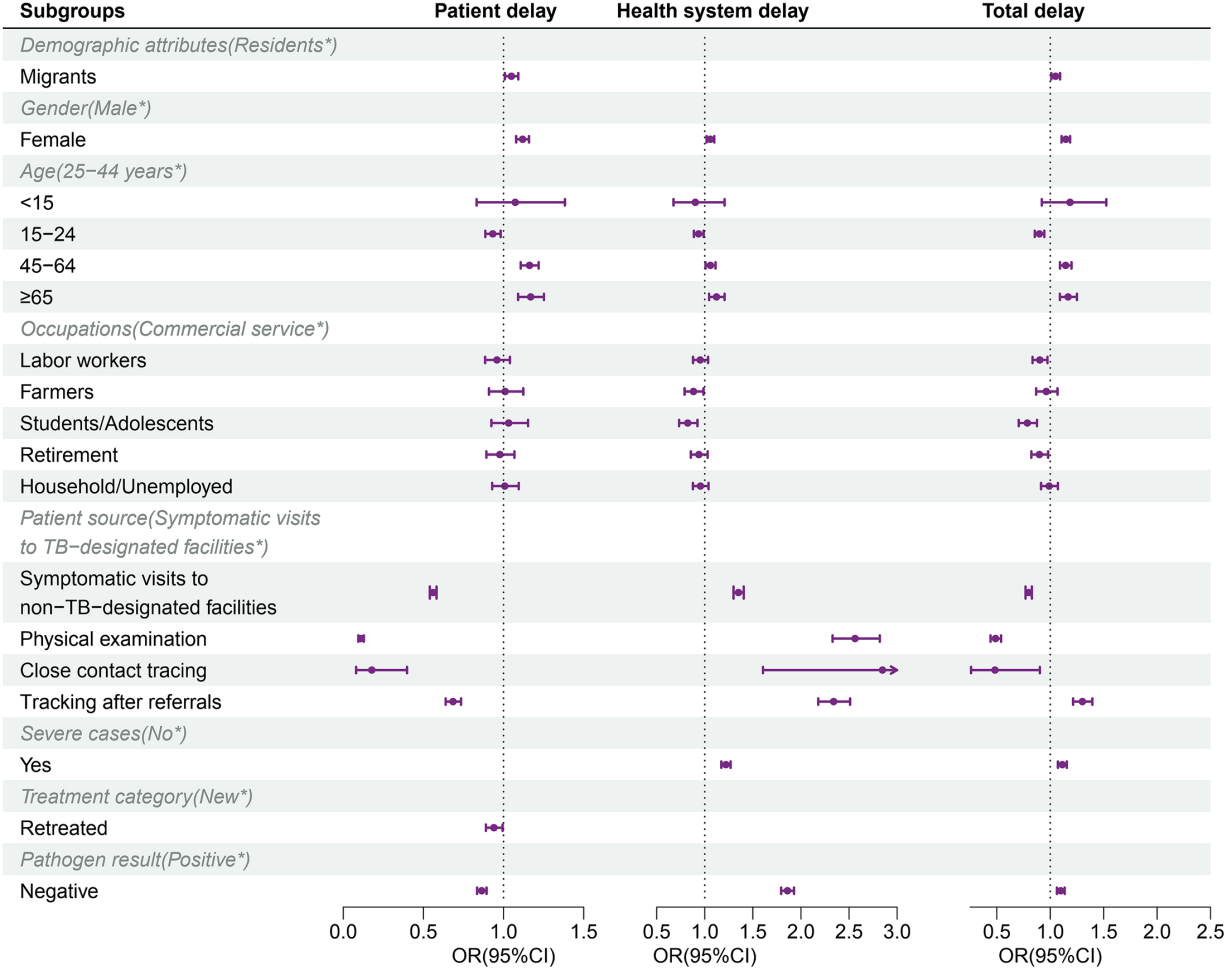
Risk factors for long patient, health system, and total delay identified by multivariable logistic regression. *Reference category. OR, odds ratio; CI, confidence interval.

After performing the univariate analysis (Supplementary Table S1), the final multivariable logistic analysis result (Figure 3; Supplementary Table S2) showed individual risk factors that were independently associated with long delay (LPD, LHD, and LDD). Compared to residents, migrants were significantly associated with LPD (aOR, 1.048, 95% CI: 1.007–1.090) and LDD (aOR, 1.052, 95% CI: 1.010–1.094). Students/ Adolescents patients were independent protective factors for LHD (aOR, 0.833, 95% CI: 0.739–0.932) and LDD (aOR, 0.788, 95% CI: 0.700–0.870). For patient source, compared with symptomatic visits to TB-designated facilities, symptomatic visits to non-TB-designated facilities (aOR, 0.559; 95CI, 0.539–0.581), physical examination (aOR, 0.110; 95CI, 0.094–0.128), close contact tracing (aOR, 0.178; 95CI, 0.080–0.399), and tracking after referrals (aOR, 0.685; 95CI, 0.639–0.734) were protective factors for LPD, i.e., shorter patient delay, however they were risk factors for LHD. For pathogen results, pathogen-negative was significantly associated with shorter patient delay (aOR, 0.864; 95CI, 0.835–0.894), while increased health system delay (aOR, 1.859; 95CI, 1.793–1.927). In addition, retreated TB patients were protective factors for LPD (aOR, 0.940; 95CI, 0.890–0.994), and severe cases were significantly associated with the longer health system (aOR, 1.215; 95CI, 1.172–1.265) and total delays (aOR, 1.111; 95CI, 1.070–1.153).

### 3.4 Spatial heterogeneity of TB diagnostic delay in Shanghai

The spatial distributions of median delay days for TB patients at the county level are shown in Supplementary Figure S3. The median days of total delay were higher in the eastern counties of Shanghai and Chongming Island. The median days of health system delay were higher in western counties, including the Songjiang and Minhang districts of Shanghai, with a median delay of more than 12 days in most counties in these districts, which had a high proportion of internal migrants and industrial parks.

**Figure 4:**
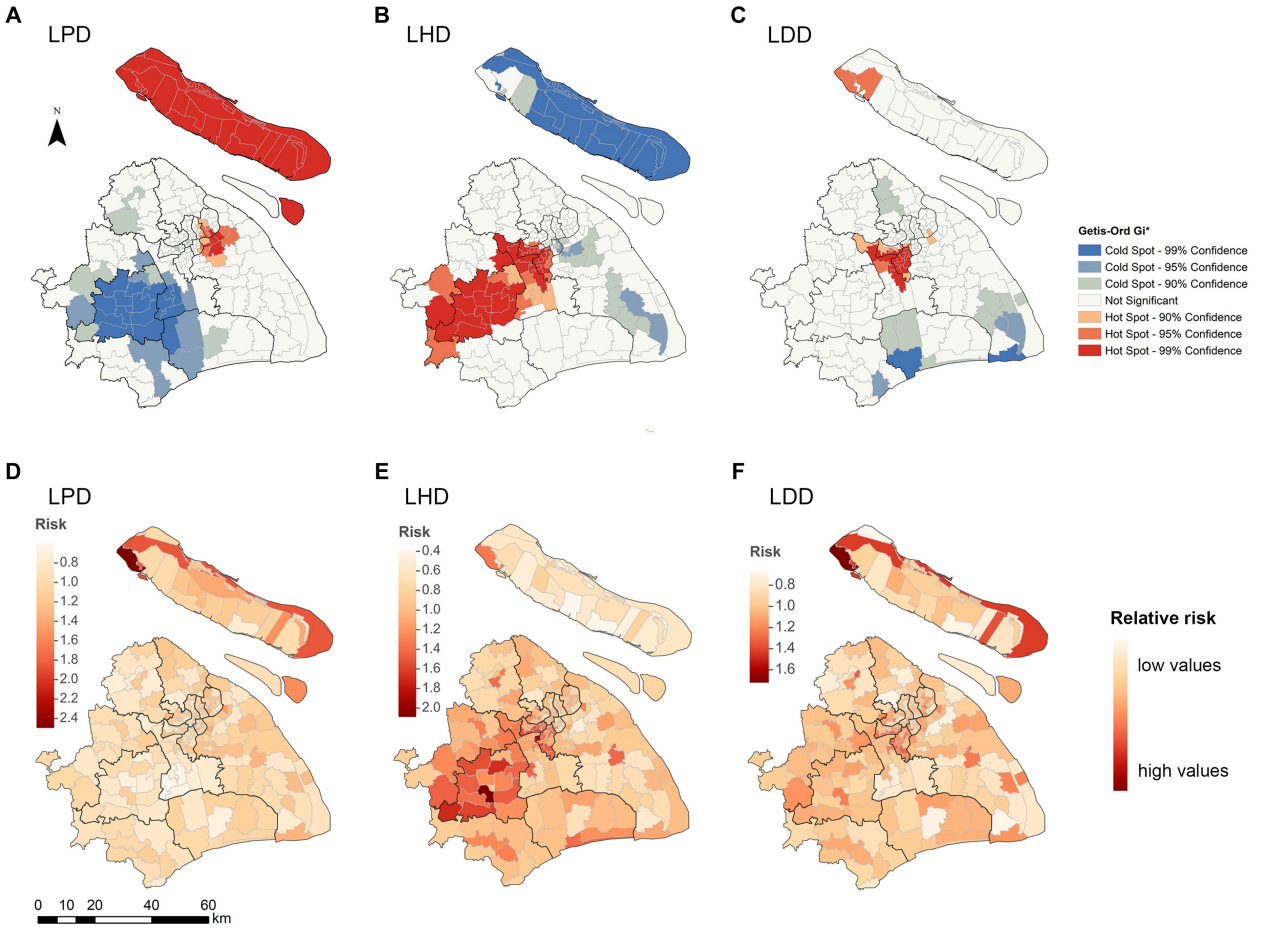
Spatial heterogeneity of long delays among TB patients by county level in Shanghai, 2010–2018. **(A–C)** Spatial clustering patterns of long delays by county level. The Getis-Ord Gi* method identified the hot and cold spots with different colors. **(D–F)** The posterior relative risk of long delays with the Hierarchical Bayesian model. LPD, long patient delay; LHD, long health system delay; LDD, long diagnostic delay.

To further identify the spatial heterogeneity of long-delayed areas in Shanghai, we conducted the spatial hotspot analysis using Getis-Ord Gi*. The results showed statistically significant hotspot areas for LPD/LHD/LDD (Figures 4A–C). There were 24 high-clustering counties of LDD, most located in the central counties of Shanghai. For LPD, we detected 33 high-clustering counties of overall patients, mainly located in Chongming Island and some counties in Yangpu and Pudong Districts. For LHD, we detected 46 high-clustering counties, mainly located in western areas, which have a higher concentration of migrants and industrial parks. The hotspot areas of LPD and LHD for both resident and migrant patients were highly consistent with those for overall patients, the hotspot areas for LPD and LHD were located in Chongming Island and the western areas, respectively (Supplementary Figures S4, S5).

### 3.5 High delay areas and spatial factors using Bayesian spatial analysis

The hierarchical Bayesian spatial analysis revealed the spatial factors associated with longer delay (Table 2). We found that industrial parks (RR, 1.610, 95% CI: 1.115–2.331), migrants (RR, 1.204, 95% CI: 1.048–1.375), household size (RR, 3.552 95% CI: 1.278–9.755), and TB notification rates (RR, 1.288, 95% CI: 1.051–1.572) were the risk factors associated with the increase of LPD in Shanghai. In contrast, the sex ratio was associated with reduced LPD (RR, 0.110, 95% CI: 0.020–0.575), indicating that areas with a higher proportion of males were less likely to have LPD. The risk factors for LHD were industrial parks (RR, 1.596, 95% CI: 1.089–2.335) and TB notification rates (RR, 1.291, 95% CI: 1.049–1.585). The sex ratio showed a significant negative effect on LHD (RR, 0.117, 95% CI: 0.020–0.633). Meanwhile, the risk factors for LDD were industrial parks (RR, 1.590, 95% CI: 1.099–2.309), migrants (RR, 1.204, 95% CI: 1.039–1.387), household size (RR, 3.419, 95% CI: 1.158–10.016), and TB notification rates (RR, 1.293, 95% CI: 1.055–1.590), with males associated with reduced LDD (RR, 0.096, 95% CI: 0.019–0.495).

**Table 2:** The hierarchical Bayesian spatial analysis results.

Parameter	Patient delay		Health system delay		Total delay	
	RR	95%CI	RR	95%CI	RR	95%CI
Presence of industry park	1.610	(1.115, 2.331)	1.596	(1.089, 2.335)	1.590	(1.099, 2.309)
Percentage of Migrants (per 10% increase)	1.204	(1.048, 1.375)	1.142	(0.983, 1.348)	1.204	(1.039, 1.387)
Population density (per 1,000 increase)	1.008	(0.990, 1.025)	1.006	(0.987, 1.025)	1.007	(0.988, 1.024)
*Per capita* GDP (per 1,000 increase)	1.002	(0.961, 1.041)	0.976	(0.936, 1.018)	0.992	(0.952, 1.031)
Household size (number of individual)	3.552	(1.278, 9.755)	3.060	(0.979, 9.140)	3.419	(1.158, 10.016)
Total Sex ratio (female as the reference)	0.110	(0.020, 0.575)	0.117	(0.022, 0.633)	0.096	(0.019, 0.495)
Percentage of elder population (per 10% increase)	1.661	(0.216, 11.887)	0.407	(0.045, 4.589)	0.911	(0.111, 7.102)
TB notification rates (/100,000 population)	1.288	(1.051, 1.572)	1.291	(1.049, 1.585)	1.293	(1.055, 1.590)
Health Staff (/10,000 population)	0.951	(0.900, 1.008)	0.992	(0.934, 1.052)	0.969	(0.916, 1.026)

RR, relative risk.

In addition, Bayesian spatial disease mapping was used to identify regions with increased risk of LPD, LHD, and LDD (Figures 4D–F). The high-risk areas with RR greater than one for LPD were further concentrated in Chongming Island. For LHD, they were concentrated in the western district, with a high proportion of migrant people and patients, as well as the industrial parks.

## 4 Discussion

Our study comprehensively investigated the TB diagnostic delay in Shanghai from 2010 to 2018. The declining trend of LHD since 2015, both in migrant and resident TB patients, echoes the scale-up of implementation of GeneXpert in the whole city, suggesting that applying rapid diagnostic tools may have reduced health system delays. Of note, we identified a significant social-demographic heterogeneity in diagnostic delay, necessitating targeted strategies to reduce further delays among high-risk population groups, including migrants. Diagnostic delays also showed significant spatial heterogeneity, with Chongming Island as an LPD hotspot area and the western districts as an LHD hotspot area. Furthermore, active case finding can significantly reduce patient delays and is an important approach for early detection of TB, such as pathogen-negative patients, thus reducing the risk of TB transmission.

Although Shanghai had equivalent delay days compared to other cities in China (11, 20, 29, 30), nearly half of TB patients experienced long diagnostic delays. This suggests that considerable efforts are still needed to reduce TB diagnosis delays. Health system delay showed a declining trend, especially after 2015. It may be attributed to the implementation of the WHO-endorsed rapid molecular diagnostic test, which has been adopted in several TB-designated hospitals in Shanghai since 2014 (31). Several reports have shown that the rapid molecular test can enhance detection sensitivity, increase bacteriological confirmation, and significantly reduce TB diagnosis delays (31–33). Therefore, it is necessary to incorporate rapid diagnostic tests more extensively into TB detection and diagnosis, identifying and addressing operational challenges to maximize the benefits of the tests, ultimately enhancing diagnostic efficiency and reducing disease transmission. Besides, a series of TB prevention and control policies and measures, along with increasing public health investments, have contributed to lowering TB diagnostic delays (34).

In our study, migrants were a risk factor for LPD. Previous studies have suggested that migrants may face more obstacles in accessing healthcare and social security (35). A survey conducted in Zhejiang Province found significant delays in seeking healthcare and diagnosis among migrants (20). These findings highlight the need to enhance health education and raise awareness about TB among migrants (20). However, the health system delays for migrants were comparable to residents, consistent with other research conducted in Shanghai (36), suggesting that Shanghai’s proactive healthcare services and policies, such as the TB-free treatment policy for migrants, have reduced barriers to diagnosis for migrants (37). Additionally, females and patients ≥65 years old were associated with longer patient and health system delays. These groups might experience more significant economic and cultural barriers, resulting in a lower willingness to seek healthcare (29). Among the older adults, TB symptoms often manifest non-specifically, increasing the difficulty in identifying and diagnosing TB (8). Besides, the older adults are more susceptible to comorbidities, hindering early TB diagnosis (38, 39). Our Bayesian spatial analysis further revealed that a larger proportion of females, older adults, and migrants were associated with high delay regions. This underscores the need to prioritize these factors related to both regional and individual levels and develop intervention measures to improve individual and population health. Retreated patients were a protective factor for LPD, potentially attributable to their enhanced TB-related knowledge (34).

Our study showed that active case finding (ACF) can shorten patient delays compared with symptomatic visits to TB-designated facilities; the latter are passive case findings that rely on patients’ self-awareness of TB-like symptoms (3). A nationwide study in China also found that ACF was the most useful method to reduce patient delay (40). ACF ensures early identification of patients who may not recognize their symptoms (41). ACF also makes health care more accessible by overcoming geographic and socioeconomic barriers. However, ACF was a risk factor for LHD. Currently, ACF is usually implemented by primary healthcare institutions, which may have relatively limited capacity to identify patients (42). Besides, studies have shown that the commonly used ACF method in China is symptom screening, which has a low sensitivity and may overlook 40–50% of TB patients, missing the opportunity for early diagnosis (43). Therefore, there is an urgent need to enhance the diagnostic capabilities of primary healthcare institutions and utilize more sensitive screening methods to maximize the role of ACF in reducing delays.

Symptomatic visits to non-TB-designated facilities also lead to longer health system delays. In our study, about one-third of the patients came through referrals, and their initial care visit facilities were usually at general hospitals (11). However, general hospitals lack the diagnostic capabilities and awareness necessary for timely TB diagnosis. Meanwhile, Chinese TB guidelines mandate that TB diagnoses must be made by TB-designated hospitals (44), further contributing to long health system delays for suspected TB patients in general hospitals. This emphasize the urgent need to strengthen the diagnostic capacity of general hospitals in China.

TB patients with LPD appeared more likely to be pathogen-positive. Delays in seeking healthcare can lead to prolonged multiplication of *Mycobacterium tuberculosis* in the body and more likely exclusion of bacterial-containing sputum (15). Diagnosis of pathogen-negative TB patients requires a comprehensive approach, leading to longer health system delays (29). Moreover, Supplementary Figure S6 shows that pathogen-negative TB patients accounted for the majority of ACF, highlighting the significance of active detection in identifying pathogen-negative patients and reducing their patient delay.

Of note, we identified a significant spatial heterogeneity in diagnostic delay. Patients on Chongming Island experienced longer patient delays, and hotspots for LPD among migrants and residents were located on Chongming Island. This scenario may be because Chongming Island is a suburban island in Shanghai, with limited healthcare resources (45). Patients on this island may encounter both temporal and spatial challenges when seeking healthcare in downtown Shanghai, which may lead to increased patient delays. Therefore, we recommend increasing the investment in Chongming’s healthcare resources, improving the local healthcare infrastructure, and enhancing active case-finding initiatives to ensure the timely identification of patients within this region.

Counties with higher TB notification rates were associated with long delay clusters, consistent with studies in other countries (12). Long-delay patients are persistent sources of TB transmission, since they continue to interact with family, communities, and colleagues, elevating the community’s TB transmission risk. A study in Shanghai also emphasized the pivotal role of diagnostic delays in TB transmission (45). This study highlights the necessity of focusing on high TB notification rate areas, which tend to exhibit longer delays, thus posing greater opportunities for transmission (46). Industrial parks and household size were risk factors for longer delay, possibly associated with low income and a lack of TB-related knowledge. Additionally, most workers in industrial parks were migrants, leading to longer delays. This suggests that implementing intervention measures in industrial parks, such as pre-employment and large-scale routine screening for migrant workers, may help reduce delays (47).

Our study has several limitations. First, we did not include economic-related and cultural-related variables, such as annual household income, individual income levels, and cultural levels, which might affect our interpretation of the factors associated with the diagnostic delays. Besides, we lack information about patient pathways before diagnosis, such as the number of medical visits to general hospitals each time. Future studies could analyze patient pathways to explore the relationship between healthcare-seeking behaviors and TB delays.

## 5 Conclusion

Although TB diagnostic delays decreased in Shanghai, long delays were still common among both migrant and resident patients. There was significant social demographic and spatial heterogeneity in TB patient delay. It is necessary to develop policies and measures targeting high-risk populations and areas with long delays, as well as to widely promote the application of rapid molecular diagnostic methods (e.g., GeneXpert). Besides, ACF can effectively reduce patient delay, and further exploration is needed to maximize the role of ACF in reducing TB delays.

## Data availability statement

The data analyzed in this study is subject to the following licenses/restrictions: the original contributions presented in the study are included in the article/Supplementary material, further inquiries can be directed to the corresponding author. Requests to access these datasets should be directed to CY, yangchg9@mail.sysu.edu.cn.

## Author contributions

RS: Data curation, Formal analysis, Methodology, Visualization, Writing – original draft. ZW: Data curation, Writing – review & editing, Formal analysis, Writing – original draft. HZ: Methodology, Formal analysis, Writing - original draft. JH: Writing – original draft. YLi: Methodology, Writing – original draft. MC: Investigation, Writing – original draft. YLv: Writing – review & editing. FZ: Writing – review & editing. YaZ: Writing – review &editing. ML: Formal analysis, Writing – review & editing. JY: Writing – review & editing. HJ: Writing – review & editing. YiZ: Writing – review & editing. JX: Writing – review & editing. YX: Writing – review & editing. JY: Writing – review & editing. YZh: Writing – review & editing. XS: Data curation, Writing – review & editing. CY: Writing – review & editing.
