# Corrigendum: Assessing heterogeneity of patient and health system delay among TB in a population with internal migrants in China

**DOI:** 10.3389/fpubh.2024.1391181

**Published:** 2024-03-05

**Authors:** Ruoyao Sun, Zheyuan Wu, Hongyin Zhang, Jinrong Huang, Yueting Liu, Meiru Chen, Yixiao Lv, Fei Zhao, Yangyi Zhang, Minjuan Li, Jiaqi Yan, Hongbing Jiang, Yiqiang Zhan, Jimin Xu, Yanzi Xu, Jianhui Yuan, Yang Zhao, Xin Shen, Chongguang Yang

**Affiliations:** ^1^School of Public Health (Shenzhen), Shenzhen Campus, Sun Yat-sen University, Shenzhen, Guangdong Province, China; ^2^Division of TB and HIV/AIDS Prevention, Shanghai Municipal Center for Disease Control and Prevention, Shanghai, China; ^3^Shanghai Institutes of Preventive Medicine, Shanghai, China; ^4^Department of Pharmacy, Beijing Hospital, National Center of Gerontology; Institute of Geriatric Medicine, Chinese Academy of Medical Sciences; Beijing Key Laboratory of Assessment of Clinical Drugs Risk and Individual Application (Beijing Hospital), Beijing, China; ^5^Department of Epidemiology, School of Public Health and Key Laboratory of Public Health Safety, Fudan University, Shanghai, China; ^6^Nanshan District Center for Disease Control and Prevention, Shenzhen, Guangdong Province, China; ^7^Department of Epidemiology of Microbial Diseases, Yale School of Public Health, New Haven, CT, United States

**Keywords:** tuberculosis, TB diagnosis delay, patient delay, health system delay, Bayesian spatial analysis

In the published article, there was an error regarding the affiliations for Chongguang Yang. The correct affiliations for Chongguang Yang are 1,6,7, not 1,5,7.

The authors apologize for this error and state that this does not change the scientific conclusions of the article in any way. The original article has been updated.

