# Supplementary material for: Assessing heterogeneity of patient and health system delay among TB in a population with internal migrants in China
Source: Front Public Health. 2024 Feb 2;12:1354515. doi: 10.3389/fpubh.2024.1354515 (PMC10869454; doi:10.3389/fpubh.2024.1354515)
Supplement: Supplementary file 1 [file Data_Sheet_1.docx]

Supplementary Material

Assessing Heterogeneity of Patient and Health System Delay among TB in a Population with Internal Migrants in China

**List for supplementary material**

**Supplementary Table S1.** The univariate logistic analysis results

**Supplementary Table S2.** Risk factors for patient health system and total delay identified by multivariable logistic regression.

**Supplementary Figure S1.** Inclusion and exclusion of patient cases for the analysis.

**Supplementary Figure S2.** Annual percentage changes of overall TB patients (A-C), resident patients (D-F), and migrant patients (G-I) with the proportion of LPD, LHD, and LDD in Shanghai, 2005–2017.

**Supplementary Figure S3.** Distribution map of median days of delays in different counties.

**Supplementary Figure S4.** Spatial clustering patterns of long delays in residents by county level in Shanghai, 2010–2018.

**Supplementary Figure S5.** Spatial clustering patterns of long delays in migrants by county level in Shanghai, 2010–2018.

**Supplementary Figure S6.** The distribution of Pathogen results stratified by different patient sources and demographic attributes.

**Supplementary Table S1.** The univariate logistic analysis results

| Characteristic | Patient delay | | | Health system delay | | | Total delay | | |
| --- | --- | --- | --- | --- | --- | --- | --- | --- | --- |
|  | OR | 95%CI | *P* value | OR | 95%CI | *P* value | OR | 95%CI | *P* value |
| **Demographic attributes** |  |  |  |  |  |  |  |  |  |
| Residents | Ref |  |  | Ref |  |  | Ref |  |  |
| Migrants | 1.048 | 1.007-1.090 | 0.022 | 1.019 | 0.974-1.06 | 0.457 | 1.052 | 1.010-1.094 | 0.014 |
| **Gender** |  |  |  |  |  |  |  |  |  |
| Male | Ref | - |  | Ref |  |  | Ref |  |  |
| Female | 1.118 | 1.079-1.159 | <0.001 | 1.058 | 1.019-1.099 | 0.003 | 1.145 | 1.105-1.186 | <0.001 |
| **Age** |  |  |  |  |  |  |  |  |  |
| 25-44 | Ref |  |  | Ref |  |  | Ref |  |  |
| <15 | 1.075 | 0.834-1.386 | 0.540 | 0.900 | 0.670-1.196 | 0.455 | 1.187 | 0.923-1.528 | 0.171 |
| 15-24 | 0.933 | 0.886-0.982 | 0.008 | 0.935 | 0.886-0.987 | 0.015 | 0.899 | 0.855-0.945 | <0.001 |
| 45-64 | 1.162 | 1.108-1.219 | <0.001 | 1.064 | 1.011-1.120 | 0.017 | 1.144 | 1.092-1.200 | <0.001 |
| ≥65 | 1.169 | 1.091-1.253 | <0.001 | 1.131 | 1.048-1.215 | 0.001 | 1.170 | 1.093-1.273 | <0.001 |
| **Ethnic group** |  |  |  |  |  |  |  |  |  |
| Han Chinese | Ref |  |  | Ref |  |  | Ref |  |  |
| Non-Han minority | 1.148 | 0.954-1.381 | 0.133 | 0.856 | 0.691-1.020 | 0.079 | 1.064 | 0.890-1.273 | 0.493 |
| **Occupations** |  |  |  |  |  |  |  |  |  |
| Commercial service | Ref |  |  | Ref |  |  | Ref |  |  |
| Labor workers | 0.960 | 0.886-1.040 | 0.314 | 0.959 | 0.876-1.032 | 0.225 | 0.907 | 0.834-0.974 | 0.014 |
| Farmers | 1.010 | 0.909-1.124 | 0.848 | 0.895 | 0.791-0.988 | 0.030 | 0.972 | 0.868-1.069 | 0.587 |
| Students/Adolescents | 1.029 | 0.921-1.150 | 0.694 | 0.833 | 0.739-0.932 | 0.002 | 0.788 | 0.700-0.870 | <0.001 |
| Retirement | 0.977 | 0.893-1.068 | 0.607 | 0.946 | 0.857-1.033 | 0.200 | 0.902 | 0.823-0.981 | 0.021 |
| Household/Unemployed | 1.008 | 0.928-1.094 | 0.854 | 0.966 | 0.878-1.039 | 0.286 | 0.998 | 0.914-1.072 | 0.801 |
| Others | 1.121 | 1.038-1.211 | 0.004 | 0.825 | 0.757-0.889 | <0.001 | 0.847 | 0.782-0.909 | <0.001 |
| Unknown | 1.263 | 1.152-1.385 | <0.001 | 1.016 | 0.923-1.118 | 0.746 | 1.185 | 1.083-1.296 | <0.001 |
| **Patient source** |  |  |  |  |  |  |  |  |  |
| Symptomatic visits to TB-designated facilities | Ref |  |  | Ref |  |  | Ref |  |  |
| Symptomatic visits to non-TB-designated facilities | 0.559 | 0.539-0.581 | <0.001 | 1.374 | 1.321-1.429 | <0.001 | 0.807 | 0.778-0.837 | <0.001 |
| Physical examination | 0.110 | 0.094-0.128 | <0.001 | 2.558 | 2.324-2.816 | <0.001 | 0.487 | 0.440-0.539 | <0.001 |
| Close contact tracing | 0.178 | 0.080-0.399 | <0.001 | 2.837 | 1.598-5.034 | <0.001 | 0.483 | 0.258-0.903 | 0.023 |
| Tracking after referrals | 0.685 | 0.639-0.734 | <0.001 | 2.332 | 2.173-2.503 | <0.001 | 1.297 | 1.212-1.390 | <0.001 |
| Others | 1.006 | 0.624-1.621 | 0.980 | 3.175 | 1.954-5.160 | <0.001 | 2.542 | 1.506-4.292 | <0.001 |
| **Severe cases** |  |  |  |  |  |  |  |  |  |
| No | Ref |  |  | Ref |  |  | Ref |  |  |
| Yes | 1.043 | 1.004-1.084 | 0.030 | 1.215 | 1.172-1.265 | <0.001 | 1.111 | 1.070-1.153 | <0.001 |
| **Treatment category** |  |  |  |  |  |  |  |  |  |
| New | Ref |  |  | Ref |  |  | Ref |  |  |
| Retreated | 0.940 | 0.889-0.994 | <0.001 | 0.996 | 0.937-1.058 | 0.886 | 0.980 | 0.927-1.035 | 0.461 |
| **Pathogen result** |  |  |  |  |  |  |  |  |  |
| Positive | Ref |  |  | Ref |  |  | Ref |  |  |
| Negative | 0.864 | 0.835-0.894 | <0.001 | 1.859 | 1.793-1.927 | <0.001 | 1.097 | 1.061-1.134 | <0.001 |
| Unknown | 1.031 | 0.926-1.148 | 0.577 | 1.026 | 0.906-1.166 | 0.670 | 0.848 | 0.761-0.944 | 0.003 |

Abbreviations: OR, odds ratio; CI, confidence interval; Ref, reference.

**Supplementary Table S2.** Risk factors for patient health system and total delay identified by multivariable logistic regression.

| Characteristic | Patient delay | | | Healthsystem delay | | | Total delay | | |
| --- | --- | --- | --- | --- | --- | --- | --- | --- | --- |
|  | OR | 95%CI | *P* value | OR | 95%CI | *P* value | OR | 95%CI | *P* value |
| **Demographic attributes** |  |  |  |  |  |  |  |  |  |
| Residents | Ref |  |  |  |  |  | Ref |  |  |
| Migrants | 1.050 | 1.008-1.092 | 0.018 |  |  |  | 1.052 | 1.012-1.094 | 0.014 |
| **Gender** |  |  |  |  |  |  |  |  |  |
| Male | Ref |  |  | Ref |  |  | Ref |  |  |
| Female | 1.119 | 1.079-1.159 | <0.001 | 1.058 | 1.019-1.100 | 0.003 | 1.146 | 1.106-1.186 | <0.001 |
| **Age** |  |  |  |  |  |  |  |  |  |
| 25-44 | Ref |  |  | Ref |  |  | Ref |  |  |
| <15 | 1.073 | 0.832-1.383 | 0.587 | 0.906 | 0.678-1.210 | 0.487 | 1.185 | 0.921-1.525 | 0.187 |
| 15-24 | 0.933 | 0.886-0.982 | 0.008 | 0.936 | 0.887-0.988 | 0.016 | 0.899 | 0.855-0.945 | <0.001 |
| 45-64 | 1.162 | 1.107-1.219 | <0.001 | 1.059 | 1.008-1.112 | 0.022 | 1.143 | 1.091-1.199 | <0.001 |
| ≥65 | 1.169 | 1.091-1.252 | <0.001 | 1.122 | 1.045-1.206 | 0.002 | 1.167 | 1.090-1.250 | <0.001 |
| **Occupations** |  |  |  |  |  |  |  |  |  |
| Commercial service | Ref |  |  | Ref |  |  | Ref |  |  |
| Labor workers | 0.959 | 0.885-1.040 | 0.312 | 0.962 | 0.887-1.044 | 0.248 | 0.907 | 0.834-0.974 | 0.009 |
| Farmers | 1.010 | 0.909-1.123 | 0.850 | 0.894 | 0.790-0.999 | 0.028 | 0.971 | 0.868-1.068 | 0.478 |
| Students/Adolescents | 1.032 | 0.924-1.153 | 0.579 | 0.827 | 0.735-0.928 | 0.001 | 0.786 | 0.705-0.876 | <0.001 |
| Retirement | 0.977 | 0.893-1.068 | 0.609 | 0.943 | 0.855-1.035 | 0.183 | 0.901 | 0.823-0.980 | 0.016 |
| Household/Unemployed | 1.008 | 0.929-1.095 | 0.842 | 0.966 | 0.877-1.051 | 0.284 | 0.998 | 0.914-1.072 | 0.793 |
| Others | 1.121 | 1.038-1.211 | 0.004 | 0.824 | 0.757-0.893 | <0.001 | 0.844 | 0.782-0.909 | <0.001 |
| Unknown | 1.263 | 1.152-1.385 | <0.001 | 1.016 | 0.923-1.118 | 0.749 | 1.184 | 1.082-1.296 | <0.001 |
| **Patient source** |  |  |  |  |  |  |  |  |  |
| Symptomatic visits to TB-designated facilities | Ref |  |  | Ref |  |  | Ref |  |  |
| Symptomatic visits to non-TB-designated facilities | 0.561 | 0.540-0.582 | <0.001 | 1.375 | 1.322-1.430 | <0.001 | 0.808 | 0.779-0.838 | <0.001 |
| Physical examination | 0.110 | 0.094-0.128 | <0.001 | 2.558 | 2.329-2.815 | <0.001 | 0.488 | 0.441-0.540 | <0.001 |
| Close contact tracing | 0.178 | 0.080-0.398 | <0.001 | 2.845 | 1.605-5.049 | <0.001 | 0.483 | 0.259-0.903 | 0.023 |
| Tracking after referrals | 0.685 | 0.639-0.735 | <0.001 | 2.335 | 2.180-2.506 | 0.001 | 1.299 | 1.212-1.391 | <0.001 |
| Others | 1.013 | 0.629-1.633 | 0.957 | 3.149 | 1.939-5.114 | 0.001 | 2.539 | 1.504-4.286 | <0.001 |
| **Severe cases** |  |  |  |  |  |  |  |  |  |
| No |  |  |  | Ref |  |  |  |  |  |
| Yes |  |  |  | 1.214 | 1.167-1.264 | <0.001 | 1.110 | 1.069-1.152 | <0.001 |
| **Treatment category** |  |  |  |  |  |  |  |  |  |
| New | Ref |  |  |  |  |  |  |  |  |
| Retreated | 0.940 | 0.890-0.994 | 0.030 |  |  |  |  |  |  |
| **Pathogen result** |  |  |  |  |  |  |  |  |  |
| Positive | Ref |  |  | Ref |  |  | Ref |  |  |
| Negative | 0.864 | 0.835-0.894 | <0.001 | 1.861 | 1.794-1.928 | <0.001 | 1.099 | 1.063-1.136 | <0.001 |
| Unknown | 1.030 | 0.925-1.147 | 0.590 | 1.027 | 0.907-1.165 | 0.657 | 0.849 | 0.762-0.945 | 0.003 |

Abbreviations: OR, odds ratio; CI, confidence interval; Ref, reference.


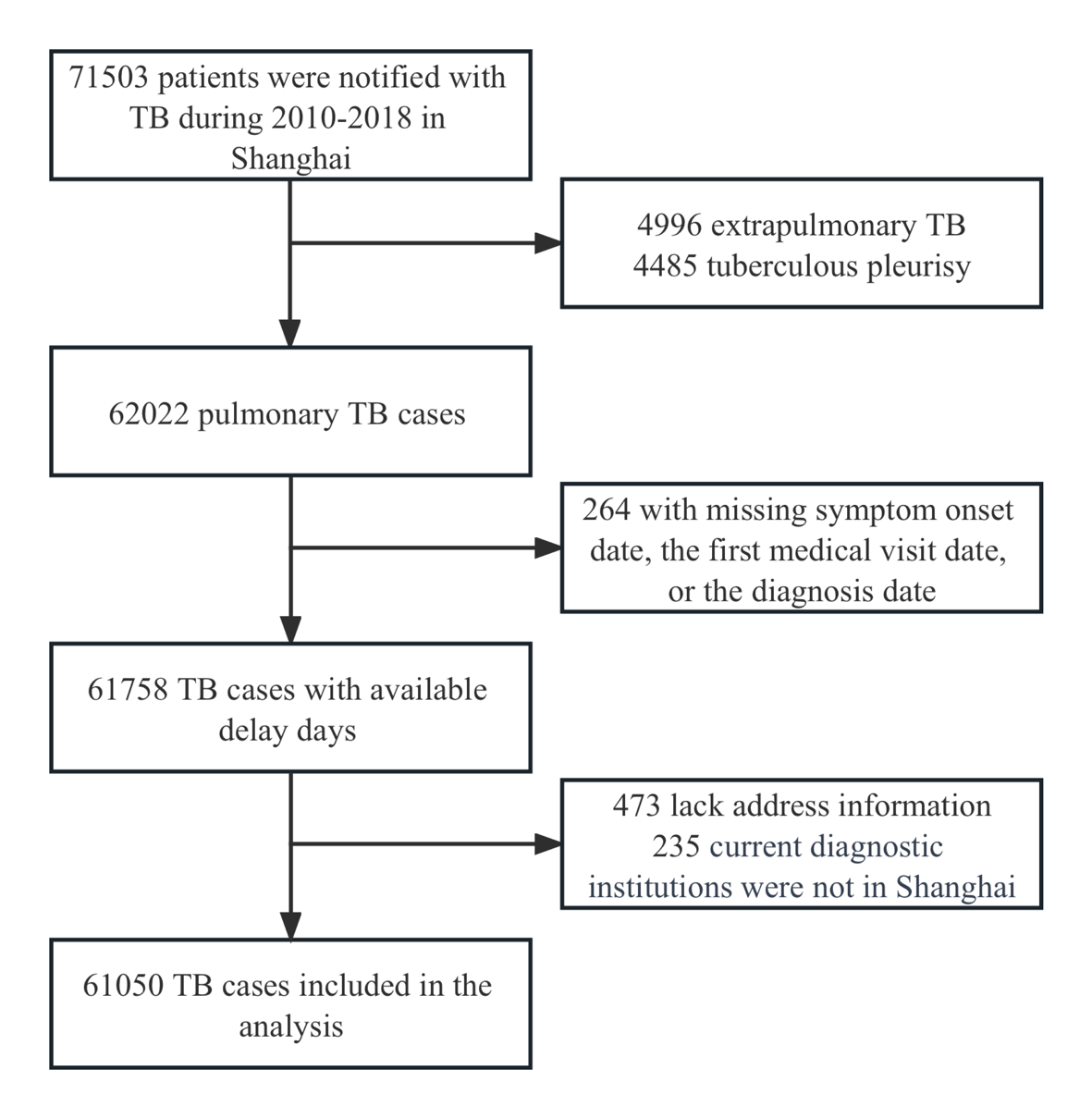


**Supplementary Figure S1.** Inclusion and exclusion of patient cases for the analysis

Abbreviations: TB, tuberculosis


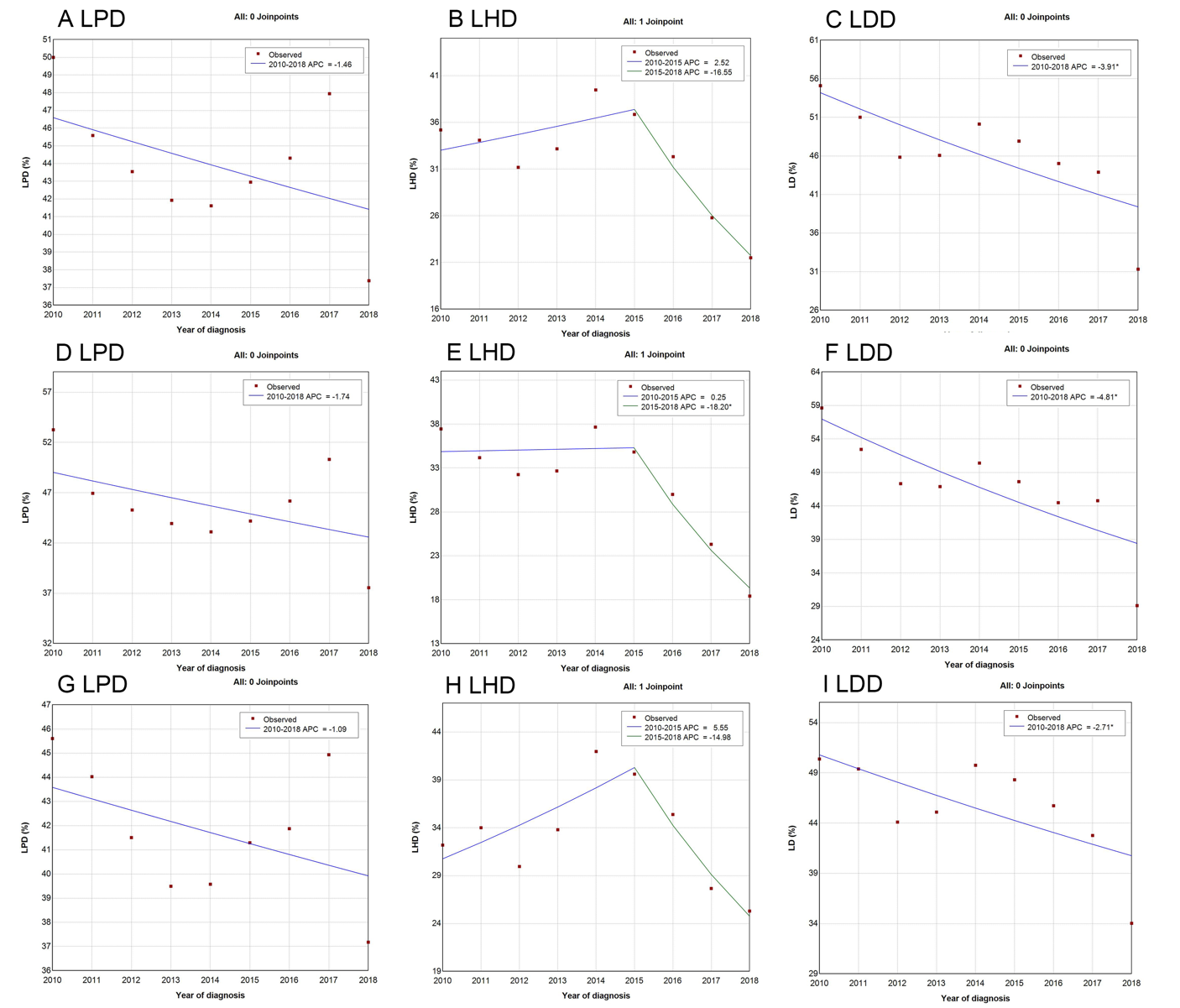
 **Supplementary Figure S2.** Annual percentage changes of overall TB patients (A-C), resident patients (D-F), and migrant patients (G-I) with the proportion of LPD, LHD, and LDD in Shanghai, 2005–2017. A.LPD; B.LHD; C.LDD. *Indicate that the Annual Percent Change (APC) is significantly different from zero at the alpha = 0.05 level.

Abbreviations: APC, annual percent change; LPD: long patient delay; LHD: long health system delay; LDD: long diagnostic delay.


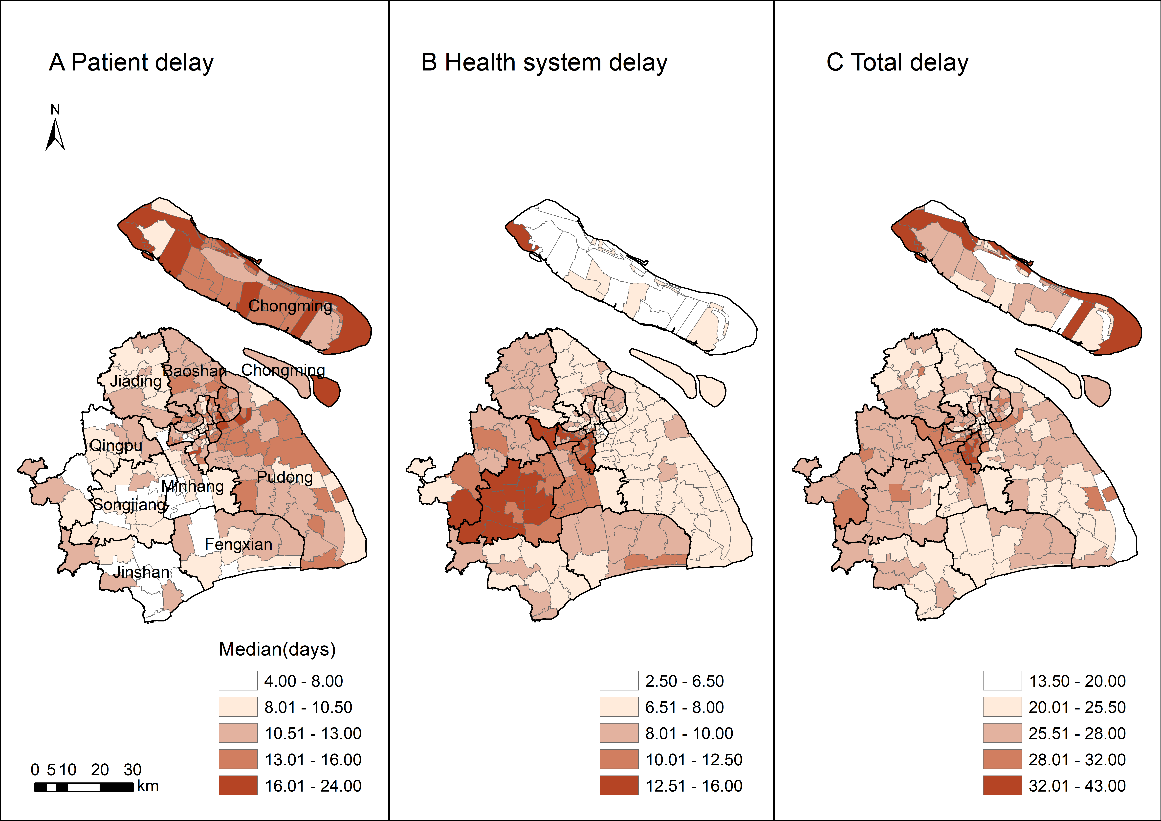


**Supplementary Figure S3.** Distribution map of median days of delays in different counties. A. Patient delay; B. Health system delay; C. Total delay.


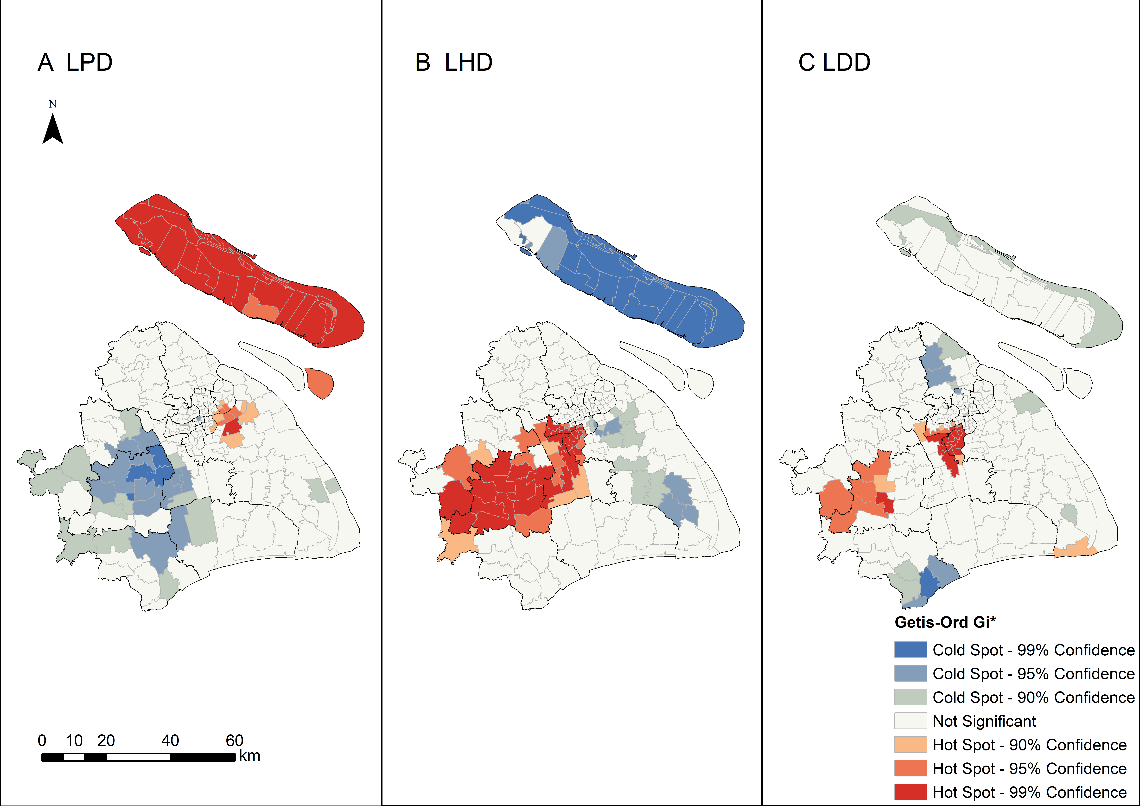


**Supplementary Figure S4.** Spatial clustering patterns of long delays in residents by county level in Shanghai, 2010–2018. A.LPD; B.LHD; C.LDD.

Abbreviations: LPD: long patient delay; LHD: long health system delay; LDD: long diagnostic delay.


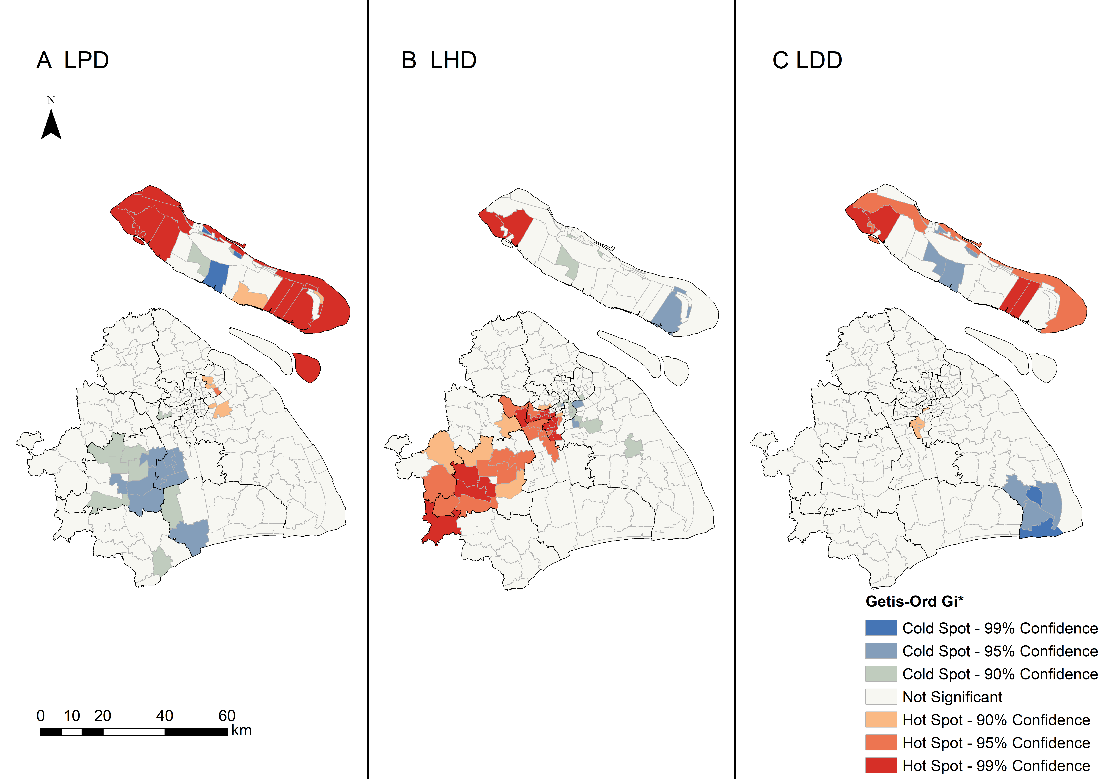


**Supplementary Figure S5.** Spatial clustering patterns of long delays in migrants by county level in Shanghai, 2010–2018. A.LPD; B.LHD; C.LDD.

Abbreviations: LPD: long patient delay; LHD: long health system delay; LDD: long diagnostic delay.


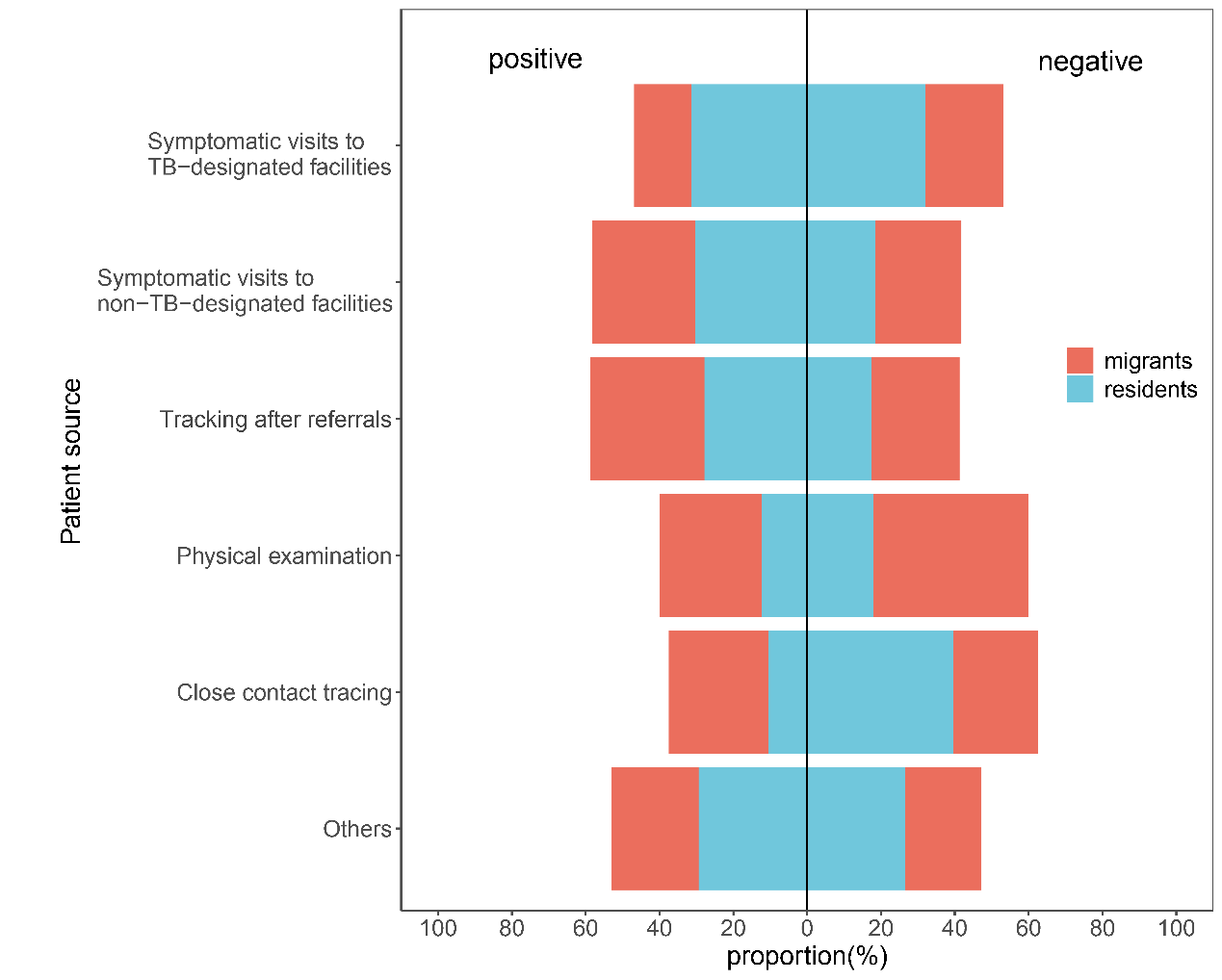


**Supplementary Figure S6.** The distribution of Pathogen results stratified by different patient sources and demographic attributes.
